# What are the needs of people with dementia in acute hospital settings, and what interventions are made to meet these needs? A systematic integrative review of the literature

**DOI:** 10.1186/s12913-020-05618-3

**Published:** 2020-08-07

**Authors:** Janne Røsvik, Anne Marie Mork Rokstad

**Affiliations:** 1 Norwegian National Advisory Unit on Ageing and Health, Vestfold Hospital Trust, Tønsberg, Norway; 2 Department of Geriatric Medicine, Oslo University Hospital-Ullevål, Oslo, Norway; 3 Faculty of Health Sciences and Social Care, Molde University College, Molde, Norway

**Keywords:** Dementia, Acute hospital, Needs, Interventions, Person-centred care, Training, Organisation, Physical environment

## Abstract

**Background:**

Research aiming to improve the hospital experience for patients with dementia and their informal carers is strongly recommended. The present review aimed to describe the research on interventions to meet the needs of people with dementia in acute hospital settings regarding physical environment, organization of care, and staff knowledge of dementia and competence in person-centred care. An integrative review design was applied. We searched for studies in PubMed, Ovid Medline, Cinahl, Embase, Swemed+, and Cochrane databases using the Mixed Methods Appraisal Tool (MMAT) for quality evaluation. Twenty-seven articles were included, describing the perspectives of people with dementia, informal carers, and professional carers. The MMAT score ranged from two to four. Twelve studies described needs and experiences, mostly using a qualitative design. Common themes and results were synthesized. The studies identified a need to enhance staff competence regarding dementia and person-centred care. Fifteen studies described interventions: two were qualitative; three used mixed method, and 10 were quantitative, of which two were randomized controlled trials and eight were observational studies. Five types of interventions were identified. Three types could positively impact staff knowledge about dementia and person-centred care. One type was experienced as positive regarding organisation of care for patients with dementia. None of the intervention studies found evidence for effects on the identified needs regarding physical environment.

**Conclusion:**

The included studies suggest that staff need more knowledge regarding dementia and person-centred dementia care and that training interventions implemented to enhance staff competence had promising results. However, there is a need for research on the needs of patients with dementia in acute hospital settings regarding physical environment and effect of design elements. There is also a scarcity of intervention studies focusing on the effect of models of care that support the psychosocial needs of patients with dementia.

## Background

Admission to an acute hospital can be both confusing and frightening for a person with dementia. Even though they are in need of hospital treatment, the stay might have negative impact on their physical, mental and cognitive abilities [1, 2]. As stated by the World Alzheimer’s Report 2016, there are significant gaps in health care service research regarding the hospital environment for people with dementia. Research aiming to improve the hospital experience for this group of patients and their informal carers is strongly recommended [3]. Hospitalization of people with dementia often leads to an increase in behavioural and psychological symptoms in dementia, risk of poor outcomes, higher incidence of harm, and further cognitive decline [1, 2, 4]. A prospective cohort study of 10,014 hospital admissions revealed that among people with a dementia diagnosis, delirium occurred in 45.8% during the hospital stay [5]. Consequences for people with dementia admitted to hospital include higher mortality rates, increased likelihood of falls, functional decline, spatial disorientation, possible malnutrition and dehydration, increased reliance on caregivers, depression, and delirium [1]. Additionally, they may experience more pain, thirst, fear, and over-stimulation than people without a cognitive impairment while in hospital, partly due to their impaired ability to communicate [6]. In acute hospitals the level of activity is high, therefore, monitoring and managing patients’ acute needs is a first priority for staff [7].

The concept ‘dementia-friendly hospital’ is linked to the elements focused on in the concept of ‘elder-friendly hospitals’: social climate, policy and procedures, care systems and processes, and physical design [8]. Both principles for universal design [9] and for dementia-friendly design [10] have been used to guide new construction and the refurbishment of existing hospitals. Universal access or design means to design and compose an environment so that it can be “accessed, understood and used to the greatest extent possible by all people regardless of their age, size, ability or disability” [9]. The scoping review of Parke and colleagues (2017) focused on the impact of design and architectural features on the independent function of hospitalized older people with dementia. They concluded that intervention studies are lacking in this area and recommended physical design changes to produce positive impacts on people with dementia in an acute care setting [11].

Several countries have developed national guidelines stating that person-centred care should be the basis of care for people with dementia [12, 13]. The most widely used and recognized person-centred care approach is the care philosophy of social psychologist Tom Kitwood [14]. This philosophy has four key elements: 1) valuing people with dementia and those who care for them, 2) treating people as individuals, 3) looking at the world from the perspective of the person with dementia, and 4) a positive social environment in which the person living with dementia can experience relative well-being [15]. Like the dementia-friendly hospital concept, this philosophy underlines that the physical and social environment should support people with dementia. Providing person-centred care implies to identify and respond to the individual needs and preferences of the person with dementia, their carer(s) and family. The meta-synthesis of Turner and colleagues (2017) underlined the importance of person-centred care for people with dementia in general hospitals, pointed out a gap between actual and desired practice, and highlighted a need for more education and training [16]. Fessey’s (2007) study described a lack of understanding of person-centred care among hospital nursing staff and found that application of such knowledge in practice was heavily influenced by the care environment [17].

The need for staff competence and skills in dementia care is underlined by informal caregivers. A systematic review made by Beardon and colleagues (2018) summarized informal carers’ experiences of acute hospital care aiming to inform best practice service delivery. According to the informal caregivers, there is a need for improvement in staff training to develop staff’s capability to provide help with personal care needs and to safeguard older patients’ dignity [18].

The aim of the present study was to review the documented needs of people with dementia in acute hospital settings as experienced by the patients, the informal caregivers and staff regarding the following three aspects: the physical environment, organization of care, and staff knowledge of dementia and competence in person-centred care. Furthermore, we aimed to describe the interventions made to meet these needs. The research questions were: 1) What are the needs and experiences of people with dementia as described by the patients, their informal caregivers, and staff? 2) What interventions are described? and 3) How are the needs and experiences reflected in the results of the interventions?

## Methods

An integrative literature review was found to be a suitable method as this type of review is deemed appropriate when there is change in a trend or direction of a phenomenon [19]. An integrative review summarizes past empirical or theoretical literature to provide a more comprehensive understanding of the phenomenon [20]. The approach allows for the inclusion of diverse methodologies, both empirical and theoretical, in order to present varied perspectives on the subject of concern [20–22] and promote a holistic conceptualization [19].

### Search strategy

Searches were made in PubMed, Ovid Medline, Cinahl, Embase, Swemed+, and the Cochrane databases with no constraints regarding year of publication. The search terms were selected after testing a great amount of terms. The authors cooperated with a specialized librarian in the testing. Both MeSH terms, terms used in the aim and research questions of the present study and terms found in relevant articles were used. The terms were tested in different combinations and as truncated. The search terms that resulted in relevant articles were chosen. We used the following search terms: hospital OR inpatient AND dementia OR Alzheimer* OR cognitive impairment AND physical environment / staff training OR staff education OR staff / person centred OR person centred/patient transfer* OR care pathway OR organization of care OR transitional care, screening titles and abstracts. The search was conducted March 5th 2018 and updated May 15th 2020.

### Study selection

The two reviewing authors (JR and AMMR) screened the abstracts of all papers. A data extraction form based on the inclusion and exclusion criteria was used to select studies that were relevant to include in the full-text screening. As this is an integrative review, both qualitative, quantitative, and mixed method studies were eligible for inclusion. The following inclusion criteria were used: the studies should 1) describe care for people with cognitive impairment and/or dementia in an acute hospital setting, 2) focus on the physical environment, staff competence in dementia or person-centred care, or organisation of care, 3) be published in English in a peer-reviewed publication, 4) present a clear research question or objective, and 5) describe the methods used to address the research question or objective. Studies focusing solely on discharge from hospital were excluded. Reviews, reports, commentaries, editorials, letters to the editor, and books were also excluded. The same procedure and data extraction form was used for the screening of the full texts of the selected studies. The results of the screenings were compared and reasons for disagreements were discussed and resolved.

### Quality assessment

The quality of the papers that were found to be relevant based on the full-text screening was assessed by the Mixed Methods Appraisal Tool (MMAT) [23, 24]. This screening provides a set of criteria for appraising the methodological quality of quantitative, qualitative, and mixed methods studies concomitantly. The MMAT checklist includes two screening questions that are applied across all relevant study designs. Both screening questions must be answered ‘yes’ for a paper to be qualified for inclusion and further quality screening. The studies are then systematically reviewed and rated according to how each stage of the method implementation, the quality of the sample, and the validation of the results of the different types of study designs (qualitative research, randomized controlled trials, non-randomized studies, quantitative descriptive studies, and mixed methods studies) are described. A study can achieve a score from one to four. In the current review, papers with a score of two or more were included.

To achieve inter-rater agreement, the two authors reviewed eight papers independently. The scores were compared, reasons for disagreements discussed, and agreement reached on principles for the further use of the MMAT criteria. The inter-rater agreement was 85% and considered as good. Following this initial inter-rater agreement test, the remaining papers were divided into two batches and reviewed independently by the researchers. In cases of doubt, the reviewers consulted each other and came to an agreement.

### Analysis

The analysis builds on the steps described by Whittemore and Knafl (2005) who state that the goals of the data analysis of an integrative review is “a thorough and unbiased interpretation of primary sources, along with an innovative synthesis of the evidence” (page 505) [22]. It is also pointed out that analytical methods are a poorly developed part of integrative reviews. A constant comparison method is recommended because it facilitates the distinction of patterns, themes, variations, and relationships. This implies the comparison of extracted data in order to categorize similar data. Coded categories are then compared to further the analysis and synthesis process. This approach is compatible with the integrative review’s use of data from diverse methodologies and consists of: data reduction, data display, data comparison, conclusion drawing, and verification [22].

In the present study, a table was used to present a summary of characteristics of each included article: author(s), publication year, aim, country, study design, participants, outcomes, results, conclusion and data quality score. The included papers were inspected and divided into two subgroups by a predetermined conceptual classification derived from the research questions of this review: Subgroup A consisted of studies that explored the needs and experiences of people with dementia, their informal carers, and staff in acute hospital settings. Subgroup B contained papers that described the results of intervention studies (Tables 1 and 2).

**Table 1:** Subgroup A: Needs and experiences

Author, year, country	Aim	Design	Participants	Outcomes	Findings	Conclusions	MMAT-score
Borbasi et.al 2006, Australia [25]	Explore nurses’ and health care professionals’ experiences of managing patients with dementia in hospitals	Qualitative design using semi-structured interviews	23 health care professionals with different roles and professions in 3 large teaching hospitals	In-depth subjective accounts of caring for a patient with dementia in an acute setting, Characteristics of actual practice and participants’ thoughts on best practice.	Five themes emerged: The Built Environment, The Organization System, Key Players, Current Management, Ideal Management.	Dementia raises awareness about: The risks imposed by buildings designed on the premise of the medical model, The struggle for health professionals lacking skills to provide resource-intensive dementia care, The need for an organization-wide approach to the development of best-practice principles supported by staff from the top of the organization.	4
Clissett et al. 2013, UK [26]	Explore the potential of current approaches to care in acute settings to enhance personhood in older adults with dementia	A qualitative design using non-participant observations of care and interviews after discharge concerning the experiences of patients with cognitive impairment	29 patients with cognitive impairment	The current experiences of people with dementia, family carers, and co-patients during hospitalisation for acute illness	Health care professionals in acute settings were not taking advantage of all opportunities to sustain personhood for people with dementia.	There is a need for the concept of person-centred care to be valued at the level of both the individual and the organisation/team for people with dementia to have appropriate care in acute settings.	4
Ernst et al. 2019, Switzerland [27]	Investigate health professionals’ (HP) care provision to persons with cognitive impairment and associated challenges	A concurrent, cross-sectional mixed method design. Online survey and 4 focus group interviews	339 (HP) working in acute geriatrics wards and general internal medicine wards in 2 urban hospitals: registered nurses, physicians, nurse assistants, social workers, therapists, dieticians, and others	Extent to which HP perceived their care provision to be person-centered and evidence-based, and experience distress in looking after this patient group. HP’s experience of care provision	More than half of the HP reported to act always or very frequently in person-centered and evidence-based ways, and 2/3 experienced challenging behaviors as moderately to very distressing. HP working in acute geriatric wards demonstrate statistically significant higher levels of person-centered and evidence-based care provision, and lower distress. Their caring practices pertained to building a relationship, addressing specific needs, involving family members, and working collaboratively	Findings suggest that geriatric models of care delivery support staff in meeting the needs of persons with cognitive impairment. HP require an acute care culture that values relational, collaborative and coordinated care as essential to patient safety and quality of care and supports the consistent implementation of evidence-based practices for this patient group.	3
Hung et al. 2017, Canada [28]	To explore hospital environment from the perspectives of patients with dementia	Qualitative action research design using go-along interviews, video recording, and participant observation	5 participants (3 men and 2 women) aged 65–84 with a diagnosis of dementia	Opinions and perspectives of patients with dementia about the hospital environment	Four interlinked themes: A place of enabling independence, A place of safety, A place of supporting social interactions, A place of respect.	Patient participant provided useful insights and pointed out practical solutions for improvement.	4
Jensen et al. 2019, Denmark [29]	To investigate how oral medicine was administered to hip fracture patients with Alzheimer’s disease during acute hospital stay	Qualitative design using participant observation as a passive observer	3 patients with Alzheimer’s disease aged 87–95 years	Activities related to caring	Two major themes: Concealed medication, Dialogue and engagement on medicine intake.	Careful handover of information on person-centred dementia care can play an important role in making hospital stays more dementia friendly.	4
Jensen et al. 2019, Denmark [30]	Investigate nurses’ experiences of caring for people with dementia	Qualitative interviews. Hermeneutic phenomenological research methods	8 nurses with various levels of expertise in an acute orthopaedic ward	Nurses’ experiences	Two major themes with sub-themes: Nurse communication and patient information: -Drowning in the electronic patient record Somatic priority -Hospital environment -Care compromise: preconceived ideas and frustrations -Calm and adaptive -Sentiment and willingness to learn -Variations to standardised care	Orthopaedic nurses should work to adopt a positive attitude, and person-centred approach, towards dementia care. Electronic patient record should be supplemented by oral dissemination to some extent, as information, plans of action and knowledge about the care situation has a tendency to drown in chronological data presentation	4
Kelley et al. 2019, UK [31]	Explore how family involvement impacts upon experiences of hospital care for people living with dementia	A qualitative ethnographic study using observations, conversations and interviews over two 7–9 month periods	12 dyads of people living with dementia and their families and staff on 2 care of older people acute hospital wards in 2 cities: a rehabilitation ward and a general hospital ward	Experiences of hospital care for people living with dementia	Patients could experience a lack of connection on multiple levels, and long periods of time without interacting with anyone. There was great variation in the degree to which staff used opportunities to involve families in improving connections and care. When used, the knowledge and expertise of families played a crucial role in facilitating more meaningful interactions. Involvement of families and their knowledge was not routine. Care was required to ensure that family involvement did not override the needs and wishes of people living with dementia	This study demonstrates the benefits of involving families and their knowledge in care, advocating for family involvement, alongside the involvement of people living with dementia, to become a more routine component of hospital care.	3
Moyle et.al 2011, Australia [32]	To explore management of older people with dementia in an acute hospital setting	A descriptive qualitative design using semi-structured interviews with staff	13 staff working in acute medical or surgical wards in a large hospital	Experiences of staffs’ role in the care of people with dementia	The overarching theme of paradoxical care and inconsistent approaches to care emphasised safety at the expense of well-being and dignity.	Staff education and environmental resources may improve the current situation so that people with dementia receive care that takes into account their individual needs and human dignity.	2
Pinkert et al. 2018, Germany and Austria [33]	Describe nurses’ experience in caring for people with dementia in acute hospital	Qualitative secondary analysis (content analysis). Focus group discussions and expert interviews	57 nurses from 4 hospitals in Austria, 42 nurses from 5 hospitals in Germany	Nurses’ experience in caring for people with dementia	Nurses face great uncertainty in caring for people with dementia and reacts in different ways to address this uncertainty. Even for nurses who provide some form of person-centred care, the hospital environment imposes several contextual constraints. Main theme: Alterations in nursing care routines: -Sticking to routines -Becoming involved -Breaking routines -Establishing normality	Hospitals must minimise constraints to give every nurse the chance to perform person-centred care. It is important to sensitise nurses and give them sufficient training and education to enable them to care for people with dementia	3
Prato et.al. 2019, UK [34]	Explore the experiences of older adults with cognitive impairment and their relatives during an acute hospital stay	A qualitative case study design using ethnographic, non-participant observations of the patients and semi-structured interviews with their relatives and the health care staff involved in their care	6 patients with cognitive impairment, 8 relatives, and 59 members of the health care team	Experiences of older adults with cognitive impairment and their relatives during an acute hospital stay	Three themes emerged determining the quality of the hospital experience: Valuing the person, Activities of empowerment and disempowerment, and The interaction of environment with patient well-being.	Ward-based activities for patients with cognitive impairment are needed alongside a move towards care that explores measures to improve and expand relative involvement in hospital care.	4
Scerri et al. 2018, Malta [35]	Categorise the perceived and observed needs of persons with dementia and to explore whether these needs are being or have been met	Qualitative study using semi-structured interviews and observation of routine care using Dementia Care mapping	13 persons with dementia admitted in 3 acute medical wards	Participants’ experiences of their hospital stay, whether these needs were perceived to have been met.	Basic needs such as toileting, feeding etc. were not always met. The largest gap between met and unmet needs was found in patients who were either under constant observation or unable to communicate. Too much emphasis was perceived and observed to be given on what staff considered as safety needs at the expense of other needs. The patient’s need for social contact and self-esteem such as dignity and respect were often ignored and this led to patients felling devalued	Hospital staff have to be more aware of holistic needs of patients with dementia in acute settings and the way care is delivered in order to make up for these unmet needs, thus facilitating person-centred care	2
Scerri et al. 2020, Malta [36]	Explore the perceived challenges of nurse managers when caring for patients with dementia and identify possible solutions to address these challenges	Qualitative study using focus groups	16 nurse managers responsible for 11 acute medical wards	Challenges and possible solutions to address these challenges	Organizational challenges with direct impact on the quality of care were identified. Suggested solutions were realigning the hospital strategy, improving training and care coordination, redesigning the ward environment and changing leadership styles	The study highlights the complexity of improving dementia care in hospitals and continues to show that a system-wide approach is needed	3

**Table 2:** Subgroup B: Intervention studies

Autor, year, country	Aim	Intervention	Design	Participants	Outcomes	Results	Conclusion	MMAT score
Aizen et al. 2001, Israel [37]	To assess the clinical effectiveness of a hospitalization pilot project	Hospitalization with direct admittance to an acute-care geriatric unit versus through the emergency room	Retrospective comparison design using follow-up data from medical records during a 2-year period	IG: 126 nursing home residents CG: 80 nursing home residents	Length of stay, discharge disposition, mortality, cause of hospitalization, chronic medical condition, cognitive state, change of functional status during the hospital stays	No significant differences were found between groups in length of stay, mortality, or discharge disposition	Treatment of selected nursing home residents in an acute-care geriatric unit is feasible, medically effective, results in a safe discharge, and provides an alternative to transfer to an emergency room.	4
Brooke et al. 2017 UK [38]	To understand the impact of dementia-friendly ward environments on nurses’ experiences of caring for patients with dementia	Themed bays with names and colours	Qualitative design using focus groups	Junior qualified nurses (*n* = 17) and health care assistants (*n* = 21) from three wards	Perceptions and experiences of nurses working in a dementia-friendly ward environment	Four themes: ‘It doesn’t look like a hospital’- changed environment; ‘More options to provide person-centred care’- no one size fits all; ‘Before you could not see the patients’- a constant nurse presence; ‘The ward remains the same’ - resistance to change.	Facilities creating a space for social dining and activities is useful. Bay nursing supports nurses to remain present with patients. Training and education to support and engage with patients beyond the implementation of care is needed.	3
Elvish et al. 2018, England [39]	To evaluate a dementia care training programme for general hospital staff	Training for trainers and staff using manual for trainer, booklet for staff, communication skills mini guide, a card designed to stand by the hospital bedside, a PowerPoint presentation, and interview clips with people with dementia/relative	Pre–post design, cluster trial	Data from 480 staff participants for pre–post analysis	Confidence in Dementia Scale, Knowledge in Dementia Scale, Controllability beliefs scale	Significant change between pre–post training on all outcome measures	Staff knowledge in dementia and confidence in working with people with dementia significantly increased following attendance at the training sessions.	3
Goldberg et al. 2013, UK [40]	To develop and evaluate a best-practice model of general hospital acute medical care for older people with cognitive impairment	A specialist unit designed to deliver best-practice care for people with delirium or dementia staffed with medical and mental health professionals, enhanced staff training in delirium, dementia, and person-centred dementia care. Provision of organised purposeful activity and environmental modification to meet the needs of those with cognitive impairment.	Randomised controlled trial using regular unit as control condition	600 patients aged over 65 admitted for acute medical care, identified as ‘confused’ on admission	Number of days spent at home over the 90 days after randomisation. Structured non-participant observations to ascertain patients’ experiences; satisfaction of family carers with hospital care.	There was no significant difference in days spent at home between the specialist unit and standard care groups. Patients on the specialist unit spent significantly more time with positive mood and engagement and experienced more staff interactions that met emotional and psychological needs. More family carers were satisfied with care, and severe dissatisfaction was reduced.	Conclusions: Specialist care for people with delirium and dementia improved the experience of patients and satisfaction of carers, but there were no convincing benefits in health status or service use.	2
Goldberg et al. 2014, UK [41]	To compare the behaviours of staff and patients on the Medical and Mental Health Unit (MMHU) and standard care wards and provide a narrative account that helps to explain the link between structure, process, and reported outcomes.	A specialist medical and mental health unit, as described in Goldberg et al. 2013	Qualitative study design using field notes and non-participating observations (Dementia Care Mapping, DCM)	Patients aged over 65 with delirium or dementia. Median age 86, half were female.	Field notes recorded the events being observed: the behaviour and actions of the patient, the staff member, and visitors interacting with them. DCM observations of engagement, activity, and staff interactions	Cognitively impaired older patients were cared for in environments that were crowded, noisy, and lacked privacy. Staff mostly prioritised physical over psychological needs. Person-centred care was mostly delivered during activity sessions or mealtimes by activities coordinators. Mental health needs were addressed more often on MMHU than on standard care wards, but most staff time was still taken up delivering physical care.	Care provided on the MMHU was distinctly different from standard care wards. Improvements were worthwhile, but care remained challenging, and consistently good practice was difficult to maintain.	4
Naughton et al. 2005, USA [42]	To improve outcomes for cognitively impaired and delirious older adults	Multifactorial intervention. Selectively admit cognitively impaired and delirious older adults from the emergency department to an acute geriatric unit. Implement new assessment and management protocols to improve recognition and pharmacological management of cognitive impairment and delirium.	Pre-test - post-test study design	A total of 374 patients 75 years and older	Prevalence of delirium, admission to AGU, psychotropic medication use, hospital length of stay Confusion Assessment Methods Cumulative Illness Rating scale	Prevalence pf delirium was reduced at 4 months and at 9 months compared to baseline. Each case of delirium prevented saved a mean of 3.42 hospital days.	A multi-factorial intervention designed to reduce delirium in older adults was associated with less delirium and hospital savings.	4
Naughton et al. 2018, UK [43]	To measure the impact of dementia communication training plus older adult unit (OAU) placement in students’ ability to recognise opportunities for person-centred communication compared to OAU placement alone	Dementia communication training using VERA (Validation, Emotion, Reassurance, Activity) framework with follow-up reflective discussions during OAU placement	Mixed method controlled pre-post-study design using electronic survey and focus group interviews	52 students completed surveys (IG:38 and CG:14) Focus group interviews: 19 students	Students’ ability to identify person-centred responses and application of the VERA principles which was tested using case vignettes	IG: participants were significantly more likely to identify person-centred responses compared with the control group. Focus group findings: VERA was described as a flexible approach that added t participants’ communication toolkit.	The VERA framework has potential as a foundation-level dementia communication training intervention, but it requires more rigorous testing.	3
Sampson et al. 2017, UK [44]	To evaluate the impact of a systemwide training programme in dementia care for acute hospital staff	Train-the-trainer model focusing on the basic, essential competencies relevant to all sections of workforce and society. Classroom teaching, on-the-ward training, or one-to-one coaching in practice.	Mixed methods design collecting date on four levels: individual, ward, organisation, and system	1700 staff from eight acute hospital trusts	Numbers and types of staff trained, Changes in dementia care practice, Staffs’ sense of competency in dementia care (SCID).	The number of staff trained per trust ranged from 67 to 650 (total 2020). Mean SCID score increased from baseline to follow-up. Organisational level data suggested increased use of carer’s passport, delirium screening scales, and pathways. Observations demonstrated improved staff–patient interactions but little change in hospital environments.	There was a significant improvement in staffs’ sense of competence in dementia care and the quality of interactions with patients.	3
Schindel et al. 2016, Canada [45]	To investigate the impact of the education programme on acute care staff’s self-efficacy in delivering person-centred dementia care	Intervention group (IG): The Gentle Persuasive Approaches (GPA) programme focusing on person-centred care (PCC), brain changes in dementia and delirium, communication, team/patient/family debriefing, and reassurance techniques was delivered by clinician educators. Control group (CG): Waiting list group	Mixed method, nonrandomized controlled, quasi-experimental repeated measures design and focus groups	IG: 468 staff employed on 7 clinical areas at site A CG: 277 staff employed across 5 clinical areas at site B	Quantitative: scores on short version of the Self-Perceived Behavioural Management Self-Efficacy Profile descriptive statistics Qualitative: opinions, beliefs, and practices regarding dementia care and the intervention	The IG demonstrated significant improvement in self-efficacy scores from baseline to immediately postintervention, sustained at 8 weeks. No changes from baseline to 8 weeks postintervention evident in the wait-listed group. Participants described positive impacts including implementation of person-centred care approaches.	This study determined that GPA addressed the concerns expressed by staff and provided the needed knowledge and skills to manage NDB in a person-centered fashion.	3
Sinvani et al., 2018, USA [46]	To determine whether a multicomponent intervention improves care in hospitalized older adults with cognitive impairment	Multicomponent intervention including geographic unit cohorting, multidisciplinary approach, patient engagement specialists and staff education	Non-randomized controlled study using retrospective chart review with propensity score matching	952 visits (IG: 476 and CG: 476) of older adults with cognitive impairment aged > 65	In hospital mortality, LOS, discharge disposition, readmission, medication, delirium, use of restraints from medical records	Patients in the intervention group had lower in-hospital mortality, shorter stays, were less likely to have an order of constant og enhanced observation, to be taking benzodiazepines og antipsychotic medication or to have restraints compared to controls	A multicomponent intervention may offer a new paradigm in the management of older adults with cognitive impairment in hospital care	4
Spencer et al.2013, UK [47]	To examine in depth carers’ views and experiences of the delivery of patient care for people with dementia or delirium in an acute general hospital in order to evaluate a specialist Medical and Mental Health Unit (MMHU) compared with standard hospital wards	The intervention ward enhanced five aspects of care: Additional specialist staff; Staff received enhanced training in dementia, delirium, and PCC; A programme of purposeful activities were introduced; The ward environment was optimised to improve patient orientation and independence; A proactive and inclusive approach to family carers.	Qualitative study design using semi-structured interviews	40 carers of patients with cognitive impairment admitted to hospital, 20 from each of the settings	Patient admission. Carer relationship with staff. The ward environment. Patients’ daily routines. Care and medical treatment. Discharge planning.	The themes identified were related to family carers’ expectations and included activities and boredom, staff knowledge, dignity and fundamental care, the ward environment, and communication between staff and carers. Carers from MMHU appreciated improvements relating to activities, the ward environment, and staff skills, but communication and engagement with family carers were still perceived as insufficient.	Even though neither setting was perceived as wholly good or wholly bad, greater satisfaction and less dissatisfaction with care were experienced by carers from MMHU compared with standard care wards.	4
Stenvall et.al. 2011, Sweden [48]	To investigate whether a multidisciplinary postoperative intervention programme could reduce postoperative complications and improve functional recovery among people with dementia	Staff trained for individualized care planning and rehabilitation to prevent postoperative delirium. Geriatric assessment, management, and rehabilitation, including a follow-up at 4 months postoperatively. Control group: conventional postoperative routines	A randomized controlled trial	64 patients (IG: 28 and CG: 36) with a dementia diagnosis and femoral neck fracture aged > 70 years in orthopaedic acute hospital department	Postoperative complications, living situations, and functional performance during hospitalization and during follow-ups at 4 and 12 months postoperatively. Katz ADL index, Mini-Mental State Examination, The modified Organic Brain Syndrome Scale, Geriatric Depression Scale (GDS-15).	There were fewer postoperative complications in the intervention group such as urinary tract infections, nutritional problems, postoperative delirium, and falls. At 4 months a larger proportion of the intervention group had regained their previous independent indoor walking ability. At 12 months a larger proportion in the intervention group had regained ADL performance level.	Patients with dementia who suffer a hip fracture can benefit from the intervention.	3
Surr et al. 2016, UK [49]	To evaluate the efficacy of a specialist training programme for acute hospital staff regarding improving attitudes, satisfaction, and feelings of caring efficacy in provision of care to people with dementia	A cascade training programme designed for acute hospital settings focusing on person-centred dementia care and the impact of the physical environment	Repeated measures design	40 acute hospital staff working in clinical roles	Approaches to Dementia Questionnaire (ADQ), Staff Experiences of Working with Demented Residents questionnaire, Caring Efficacy Scale	A significant positive change on all three outcome measures following intermediate training compared to baseline	The results suggest that Foundation level training may be adequate for awareness raising and supporting a more positive attitude towards people with dementia. However, the findings indicate that the knowledge offered by the Intermediate level training is needed to have an impact on staff feelings of caring efficacy and satisfaction.	2
Tay et al. 2018, Singapore [50]	To evaluate the effectiveness of an acute hospital that adopts a person-centred dementia care (PCC) protocol	Intervention group (IG): moderating intrusive interventions and encouraging family members and volunteers to engage in daily activities. Control group (CG): Patients received standard medical care.	Prospective cohort study intervention versus usual care on pre-post outcomes	IG: 170 patients CG: 60 patients	Dementia type and stage, comorbidities, well-being, ill-being, functional status, agitation levels, quality of life, Charlson’s Comorbidity Index, Modified Barthel Index, Pittsburgh Agitation Scale, EuroQoL,	IG: greater gains in Modified Barthel Index function and well-being, decreased ill-being and agitation, and greater improvement in EuroQoL index score compared to CG	The findings call for wider adoption of PCC models of enhanced care for PWDs in the acute hospital setting.	4
Travers et al. 2018, Australia [51]	To evaluate the effectiveness of the collective social education process and its impact on nurses’ knowledge of dementia and screening of at-risk patients for delirium early in their admission	Nomination of a Cognition Champions leader for each ward. Inclusion of the CogChamps project at regular ward meetings. Delirium education sessions for nurses.	Quantitative descriptive study design CogChamps were assessed for demographic characteristics, knowledge, self-rated confidence and stress assessment of nurses’ delirium knowledge pre- and post-education. Audit of CAM assessments pre-and post-education	34 experienced nurses from 6 hospital wards at a large hospital	The percentage of staff that engaged in the educational sessions, nurses’ performance on knowledge tests for the accurate identification of delirium, and improvements in timely and accurate assessments of at-risk patients for delirium	Post-audit results showed a significant increase in CAM screening rates compared to baseline. Development or acquisition of resources to support nurses’ learning.	The education process led by CogChamps and supported by educators and clinical experts provides an example of successfully educating nurses about delirium and improving screening rates of patients for delirium.	3

Next, the findings of the studies in each subgroup were analysed and themes were identified (see Table 3). Data extracted from subgroup A reflected needs, conditions that were found to be of importance to meet needs, and positive and negative experiences related to the categories described in the aim of the present study (physical environment, competence of dementia and person-centred dementia care, organization and management of care). A constant comparative process was undertaken to synthesize the extracted data for each category into themes, and to verify that the emerging themes were grounded in the data of the primary sources [22]. Both authors validated the final themes in order to enhance trustworthiness.

**Table 3:** Subgroups A and B with themes and types of interventions

Categories	Themes
**Subgroup A: Needs and experiences**	
**Physical environment**	1) the importance for independence and orientation of an environment that is easy to navigate, has distinguishable features and a view of the outdoors, 2) staff’s experience of the physical environment as a barrier for patient safety.
**Organization and management of care**	1) the need for best practice principles in dementia hospital care, 2) hospital staff need more knowledge and better skills regarding dementia care, 3) staff’s experience of the agitation of patients with dementia as burdensome, 4) the need for continuity of staff to support basic psychological needs, 5) the need for social inclusion in order to feel respected, 6) the need to be consulted regarding their own care to maintain dignity, 7) the need for meaningful interaction with staff to feel safe, and 8) the importance of staff knowing patient backgrounds to enhance empowerment.
**Competence and person-centred care**	1) the importance of companionship with other patients for a positive experience of hospitalization, 2) the importance of prioritizing the needs and care of confused patients to avoid worsening dementia-related symptoms, and 3) the importance of appropriate buildings and competent staff for quality care and prevention of the use of restraints.
**Subgroup B: Types of interventions**	
1) Implementation of physical changes in the environment (*n* = 1) 2) Comprehensive train-the-trainer programmes (*n* = 4) 3) Teaching and reflection (*n* = 2) 4) Moderation of intrusive medical interventions (*n* = 1) 5) Special geriatric models (*n* = 5)	

The intervention studies in subgroup B were analysed in order to identify patterns based on the focus of the interventions, i.e. what had been done to meet the needs of patients with cognitive impairment in the hospital wards. The emerging patterns were used to classify the interventions into groups that constituted types of interventions. The results of the studies were described in a summary for each type of intervention.

For both subgroups, the perspectives included in the syntheses of results were described, i.e., whether the findings were based on the perspectives of patients with dementia, informal caregivers, staff, or all of these.

Last, a juxtaposition was made of how the identified needs and experiences were reflected in the results of the implemented interventions for each category.

The analysis process was conducted primarily by the first author with input from the second author. The analyses were discussed and validated by the two authors, who agreed on the final categorizations, themes, and interpretation.

## Results

**Fig. 1:**
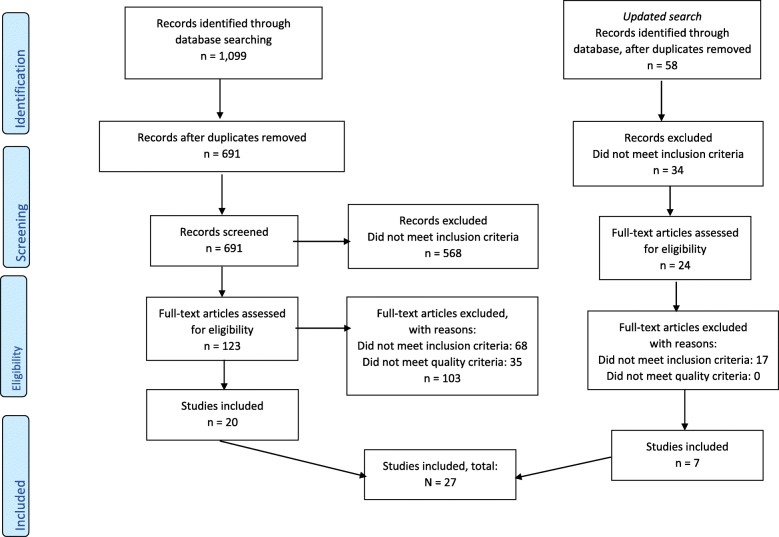
Flow diagram of the article selection process

The first search yielded 691 non-duplicate articles. Of these, 20 articles fit the inclusion criteria. Seven articles were added in the updated search. See Fig. 1 for a flow diagram of the article selection process. Of the 27 articles included, 12 were studies that described needs or experiences (subgroup A, see Table 1). Three of these studies were from the United Kingdom (UK), two from Australia, Malta and Denmark respectively. Canada, Germany/ Austria, and Switzerland had one study each. One of these studies used mixed method, the others used qualitative design. Four studies interviewed only patients with dementia. One study interviewed patients with dementia and their informal carers, one interviewed patients, informal carers and staff. Staff were interviewed in six studies. One study used observation of care, four used both observation of care and interviews with patients with dementia. The MMAT score of the studies in subgroup A ranged from 2 to 4.

Fifteen studies described interventions (subgroup B, see Table 2). Eight of these studies were from the UK, two were from USA. Singapore, Canada, Australia, Israel, and Sweden had one study each. Two studies used qualitative design; three used mixed method, and 10 were quantitative. Five types of interventions were identified: implementation of physical changes in the environment [38], comprehensive train-the-trainer programmes [44, 45, 51, 52], teaching and reflection [43, 49], moderation of intrusive medical interventions [50], and special geriatric model [37, 41, 42, 46–48]. The MMAT score of the studies in subgroup B ranged from 2 to 4.

### Physical environment

#### Needs and experiences

Eight studies described needs and experiences related to this category. The synthesis of the findings resulted in two themes: 1) *the importance for independence and orientation of an environment that is easy to navigate, has distinguishable features and a view of the outdoors, and 2) staff’s experience of the physical environment as a barrier for patient safety.*

Experiences and needs of patients with dementia related to the physical environment in acute hospital wards were described from the perspective of staff [25, 29, 33–36] persons with dementia [28, 34, 46], family carers [34] and by observations [31, 35]. The studies demonstrate the importance of an environment that enables independence for people with cognitive impairment by making it easier to navigate the surroundings. The physical environment in acute hospital wards was described as chaotic, noisy, crowded and potentially confusing for people with cognitive impairment, [29, 31, 35, 36] and a dementia friendly design with homelike environment was highly recommended for better orientation [33]. Windows were important to orientate patients in time and space [25, 34]. Distinguishable features were needed [25, 36], as some environmental conditions represented problems for patients with dementia. Bathrooms located too far from the bay area made it difficult for patients with orientation difficulties to find their way to the toilets [25]. Identical room doors and non-distinguishable hallways made it difficult for them to find their rooms. Dark areas and glare on the floor increased the risk of falling and overcrowding with equipment in the corridors made it difficult to use the handrails on the wall. This evoked feelings of danger and acute risk in the patients [28].

The acute hospital environment impacted on care delivery and staff’s attitudes and behaviours. Staff’s focus on safety and patient security in the acute hospital setting was found to be excessive, with unnatural monitoring of patients, preventing them from leaving the ward, and little emphasis on basic nursing care and meaningful interaction. Hence, the patients’ need for preserving dignity was at risk of being overlooked in the pursuit of safety as defined by staff [32, 35]. The opportunities for interaction, stimulation and orientating features facilitated by the environment was sparse, which made people with dementia less capable of making sense of the unfamiliar surroundings [31]. For the patient, safety meant not only being physically safe but also emotionally safe [28].

#### Interventions

Two types of interventions reported results related to the physical environment: 1) *implementation of physical changes in the environment, and 2) comprehensive train-the-trainer programmes in dementia care.*

The study that implemented physical changes in the environment took the following actions: organized the wards into themed bays, introduced an activity room, used pictures and distinct colours, and obstructed the view from the windows in the ward. The reason for applying opaque transfers on the windows was not explained in the article. The actions taken to improve the environment were evaluated in focus group interviews with staff. They expressed positive experiences with the intervention as they believed that the actions supported interaction with patients and relatives and increased their knowledge and understanding of them from a perspective other than the provision of clinical information. They experienced that the actions supported person-centred care. The organization of wards into themed bays increased the presence of staff, brought staff in closer contact with the patients, and created better opportunities to make running observations of the patients. However, some staff stated that the impact of the interventions on care were limited or negative [38].

The second intervention study introduced a train-the-trainer programme based on person-centred care and encouraging changes in the physical environment as part of the programme. The results assessed by observations indicated that merely encouraging trainers to focus on the physical environment and propose changes to make it fit better with the needs of patients with dementia had limited impact [44].

#### How needs and experiences are reflected in the results of interventions

The intervention *implementation of physical changes in the environment* [38] reflected the identified importance of having a view of the outdoors. The study reported that obstruction of the view had negative effects on patients’ orientation [38]. None of the other types of interventions reported results that can be used to inform decisions on making physical hospital environments dementia friendly.

### Competence of dementia and person-centred dementia care

#### Needs and experiences

The syntheses of the results of studies exploring experiences and the need for competence and person-centred dementia care in acute hospitals revealed the following list of themes: *1) the need for best practice principles in dementia hospital care, 2) hospital staff need more knowledge and better skills regarding dementia care*, *3) staff’s experience of the agitation of patients with dementia as burdensome, 4) the need for continuity of staff to support basic psychological needs, 5) the need for social inclusion in order to feel respected, 6) the need to be consulted regarding their own care to maintain dignity, 7) the need for meaningful interaction with staff to feel safe, and 8) the importance of staff knowing patient backgrounds to enhance empowerment.*

The findings in these studies were based on the perspective of staff [25, 27–30, 32–34, 36], experiences from the perspective of persons with dementia and informal carers [30, 32, 34, 35, 39], and observations [27, 32, 34, 35].

The lack of skills in hospital staff and the lack of best practice principles and guidelines were described [25, 29]. Because of staff’s generally insufficient skills in how to care for people with dementia, there was a need for better care delivery to these patients. Staff were often unable to distinguish between acute and chronic confusion and were unsure of how to assess or treat dementia and delirium. Staff expressed their frustration and concern at needing to manage agitated patients with dementia [32]. Observations of interactions between staff and patients revealed that communication could be polite and kind, but it was often functional- and task orientated. There seemed to be a priority of somatic health leading to a just-enough and just-in-time approach to the psychosocial aspects of person-centred care [29]. To meet the needs of persons with dementia for genuine connections to feel safe, staff’s communication skills should be improved to enhance meaningful interaction [31]. Examples of communications skills in dementia care are to make eye contact before you talk to the person, take into account that the person may need longer time to comprehend what is said and to respond, and act in a manner that conveys respect and creates a friendly atmosphere [34]. The key features that enhanced or diminished a sense of attachment and inclusion for persons with dementia were those of continuity of staff and staff’s communication skills. How the staff perceived the relationship with and the perspective of the patient was also important [27, 33]. Furthermore, the way staff took opportunities to engage with the patients, enhance a trustworthy relationship and demonstrate the importance of their welfare and involvement in decision making was crucial in supporting these psychological needs [26, 27, 35]. Using the preferred name to address the individual and allowing them to use their personal belongings were important for supporting people with dementia’s sense of identity. The research revealed little evidence that staff engage people with dementia in activities and support their need for occupation [35]. The lack of available activities in the hospital setting lead to boredom and frustration for people with dementia [34, 35]. Facilitating simple day-to-day activities such as going for a walk or meeting someone for conversation is essential for enhancing health and well-being by supporting feelings of social connection, independence, purpose, and a sense of belonging in the world [28]. To become involved with patients with dementia, staff must stray from familiar routines and become creative to meet individual needs [31, 33].

Additionally, limited evidence of the promotion of comfort was demonstrated. For staff, patients’ physical discomfort took precedence over emotional discomfort. There was little evidence of nursing staff viewing distressed behaviours as symptoms of discomfort. Staff tended not to act in a person-centred, proactive manner to address primary needs like hunger and thirst, nor to use sensory interventions like music or human touch to promote psychological comfort [26].

To feel respected, patients with dementia need to be socially included, however, people with dementia experienced being excluded or discriminated against due to the changes in their cognitive function. The label of dementia could lead to a feeling of being a subclass on the ward [28]. Patients with dementia were often assumed to be incapable of making care decisions. The staff tended to seek the opinion of family caregivers only, which was a serious threat to the person with dementia’s dignity and rights as the perspective of the family was not necessarily the same as that of the patient [28].

The importance of staff having life-story information to enable them to value the patient was demonstrated. However, the research revealed that staff limited their knowledge of the patients with dementia to everyday information. The staff was seen by family caregivers as mainly task orientated. This had a negative impact on the relatives’ hospital experience. Family caregivers were important agents for the empowerment of patients with cognitive impairment, and they were vital for maintaining the patients’ personhood [32, 34].

#### Interventions

Studies of interventions to improve staff’s competence in dementia care and promote person-centred dementia care in acute hospital settings included *comprehensive train-the-trainer programmes* and *teaching and reflection interventions.* In the train-the-trainer programmes, a group of trainers received courses focusing on delirium assessment [44, 51], dementia and person-centred care principles [45], and communication skills [39]. In turn, these trainers trained and supported the hospital staff. The teaching and reflection interventions were conducted as a person-centred care training programme for all hospital staff [49] and face-to-face dementia training including reflection [43]. The intervention outcomes were evaluated from the perspective of staff [2, 39, 45, 49] nursing students [43], observations [44], and post-audit results [51].

Evaluations of one train-the-trainer programme showed improvement in staff’s self-reported knowledge about dementia and an increase in their confidence in working with people with dementia [39]. Another study revealed a small increase in competence, the largest related to building relationships and initiating interaction with patients [44] as well as for delirium screening rates [51]. Furthermore, staff expressed that there was a positive impact on the implementation of person-centred care approaches, and increased effectiveness in their practice [45]. A change in staff’s interpretation and understanding of patients’ challenging behaviour was demonstrated [39]. The evaluation of the teaching and reflection intervention used to increase knowledge and enhance person-centred care indicated that the intervention can improve staff attitudes towards people with dementia and increase staff’s perceived caring efficacy [49]. Nursing students reported increased competence in connecting with patients and were significantly more likely to identify person-centred responses [43].

#### How needs and experiences are reflected in the results of interventions

Four types of interventions produced results that reflected the identified needs and experiences regarding competence and person-centred care. *Comprehensive train-the-trainer programmes* and *implementation of physical changes in the environment* demonstrated results that were relevant for the patients’ need for meaningful interaction with ward staff to experience genuine connection [38, 44]. The need for more knowledge and better skills regarding dementia care in hospital staff was reflected in the results of *comprehensive train-the-trainer programmes* and *special geriatric models* [44, 45]. Results from the latter also indicated that this type of intervention is relevant for the patients’ need for care that supports their basic psychological needs [40, 47]. *Teaching and reflection interventions* and *comprehensive train-the-trainer programmes* demonstrated results indicating positive impact on staff attitudes towards patients with dementia who were agitated [2, 39, 45, 49].

There were no results reflecting the other four identified needs and experiences in this category.

### Organisation

#### Needs and experiences

From the syntheses of findings in the studies exploring the experiences and needs related organization of health care for people with dementia in acute hospitals, the following themes emerged: *1) the importance of companionship with other patients for a positive experience of hospitalization, 2) the importance of prioritizing the needs and care of confused patients to avoid worsening dementia-related symptoms, and 3) the importance of appropriate buildings and competent staff for quality care and prevention of the use of restraints.*

The identified studies took, the perspective of staff, [25, 29, 32, 33, 36, 40] people with dementia and informal caregivers [32, 34] and included observation [32]. According to staff’s experiences, the time they spent with patients with dementia made a real difference to the patients’ well-being because vigilant and continual observation and assessment were crucial for the patient to experience safety, especially at night [25]. People with dementia and informal caregivers commented on the constraining impact of the ward routine on the patient’s experience. Being isolated in a single room without the positive impact of the company of other patients in a common area was experienced negatively. Furthermore, relatives commented on the inability of patients with cognitive impairment to adapt to the ward environment if moved around within the hospital [34]. The focus of care in hospitals was strongly geared towards acute problems, and, hence, persons with dementia were viewed as low-priority cases [32]. People with dementia were prone to be overlooked in the busy world of nurses. The inability of patients to provide a full history of events added to the complexity of care in an acute condition seemed to worsen dementia-related symptoms [25]. Fragmentation of care was demonstrated, like lack of coordination and information sharing during transitions or in planning therapeutic and diagnostic interventions [27]. Inadequate hospital systems and routines were seen as barriers for meeting the needs of people with dementia [27, 29, 31]. Furthermore, inappropriate building design and limited staff knowledge and understanding of dementia as major constraints to best practice [33], and consequently, the use of restraints was reported in each setting [25].

#### Interventions

Eight papers describing a total of seven interventions to facilitate the organisation of acute hospital services for people with dementia were identified. The interventions focused on different *special geriatric models*: organizing special geriatric units or use of comprehensive geriatric assessment [40, 41, 43, 46–48, 50]. These interventions also included staff training on dementia care. The outcomes were reported from the perspective of people with dementia [40, 48], informal caregivers [40, 47] proxy-based assessment using standardized questionnaires [37, 42, 50], observations [41, 46] and data from patient records [46].

According to observations, patients in units using special geriatric models spent significantly more time with positive moods and engagement, were able to walk around more freely, and experienced more staff interactions that met their emotional and psychological needs compared to other wards. A randomized, controlled study using a multidisciplinary postoperative intervention programme with teams applying comprehensive geriatric assessment found fewer postoperative complications in the intervention group [48]. However, most staff time was still taken up delivering physical care [41]. The studies exploring family caregivers’ satisfaction with care in special geriatric units revealed both less dissatisfaction [47] and more satisfaction with care [40] compared to standard care wards. Additionally, evaluation of special geriatric unit models revealed lower in-hospital mortality, shorter stays, less need for constant or enhanced observations, reduced use of psychotropic medication and occurrence of delirium compared to standard wards [42, 46]. Evaluation of the effectiveness of an acute hospital dementia unit adopting a person-centred care protocol moderating intrusive interventions revealed positive impacts on quality of life such as decreased agitation in people with dementia and confirmed cost-effectiveness [50]. The evaluation of the effect of using direct admittance to an acute-care geriatric unit versus an ordinary emergency room showed no impact on characteristics of the hospital stay such as length of stay, mortality, or discharge disposition [37].

#### How needs and experiences are reflected in the results of interventions

The results of implementation of *special geriatric models* and *moderating intrusive interventions* reflected the described importance of prioritizing the needs and care of confused patients. Informal carers were reported to be more satisfied with care in units implementing these types of interventions than informal carers in regular units; quality of life improved; agitation in people with dementia decreased [40, 47, 50], and there were fewer postoperative infections [48]. These types of interventions implied the need for more staff with enhanced knowledge and skills in dementia care, which is relevant for the described importance of competent staff for quality care and prevention of the use of restraints [40, 43, 47]. The importance of companionship with other patients to a positive experience of hospitalization for people with dementia was not reflected in the results of any of the types of interventions.

## Discussion

The present review found limited research regarding the needs of people with dementia in acute hospital settings and of the effect of interventions implemented to meet these needs.

Regarding the category *physical hospital environment*, all respondent groups—patients with dementia, their informal carers, and staff—underlined the importance of environmental features that helped the patients orientate in time and space. However, few intervention studies focusing on the physical hospital environment were identified, which is in line with the results of the review of Parke and colleagues (2017) [11]. Some features of a dementia-friendly hospital have been outlined [53], but it is not clear which specific physical design elements can maximize functional ability and improve independence in patients with dementia, while at the same time enhancing their safety in acute hospital units. The review of Marquardt and colleagues (2014) identified 169 studies on the impact of the design of the environment on people with dementia in long-term care facilities. According to this review, the physical environment can help people with dementia to preserve and improve their well-being, behaviour, independence, and functioning [54]. It remains to be affirmed whether corresponding physical design changes are feasible and have the same positive impact in an acute hospital ward. More research is needed to establish how design elements influence outcomes at the individual and system levels in the acute hospital. Staff differed from the other respondent groups in the present review by their focus on the barriers to patient safety represented by inappropriate buildings. Staff also deviated from the patients and informal carers regarding the category *competence in dementia and person-centred care* by not having the same focus on the psychological needs of patients with dementia. These findings correspond with earlier research which showed that staff lacked understanding of person-centred care and that application of such knowledge was heavily influenced by the care environment and high demands on staff [7, 17]. All respondent groups in the present review agreed that staff’s knowledge of dementia and communication skills needed to be enhanced, which is in line with the review of Beardon and colleagues (2018) [18]. This result may, at least partly, explain staff’s lack of focus on person-centred care. Because of insufficient knowledge about dementia, staff may not be aware that the way they communicate with the patient can influence the patients’ psychosocial needs and, in turn, the patient’s behaviour and safety. The results of the studies in this category indicate that different types of staff training interventions can enhance staff knowledge of dementia.

Patients and informal carers pointed to activities and social inclusion as important for the ability of people with dementia to express their needs and for prevention of behavioural symptoms. None of the included intervention studies reported results that were directly relevant to this finding. Lack of opportunities for social interaction has been described as a problem in previous research [18]. There is little tradition for setting aside time and resources for social activities in the acute hospital. The physical design of the hospital ward seldom facilitates such activities, which reflects a model of care that is not tuned in to the special needs of patients with dementia. The results of the studies in the category *organization* showed that the needs and care of confused patients were not prioritized in acute hospital wards; the wards were strongly geared towards acute medical problems. As the number and percentage of patients with dementia increase, adopting a form of organisation that takes their special need for social inclusion and activity into account should be considered as this may promote well-being and prevent agitation. Competent staff was a key element in the special care model interventions of the included studies, which reported positive effects on psychotropic medication use (reduction), delirium, and informal carers’ satisfaction with care. However, not even these models had resources such as staff, time, space, and equipment designated to support the need of patients with dementia for activity and social inclusion. The present review found no studies that evaluated a form of organization or model of care designed to prevent behavioural symptoms of dementia by implementing social activities in an acute hospital ward.

### Methodological considerations

Only papers reported in English were included, therefore, information regarding hospital care for people with dementia reported in other languages is not accounted for.

The present review does not include studies on discharge and transfer from hospital to community care or other health care services. These aspects of the hospitalization process are important for patients with dementia and their informal carers. However, this review describes three extensive areas, adding a fourth area would make the scope too broad for one review.

The narrow range of scores on the MMAT, two as the lowest accepted score, four the maximum score, illustrates the limitation of this tool in differentiating the quality of the included studies. However, the MMAT permits concomitant appraisal and description of the methodological quality in qualitative, quantitative, and mixed-method studies, which was considered a strength in the current review.

Almost equal numbers of quantitative and qualitative studies were included, which gives a broad view of the situation regarding needs, experiences, and effects.

## Conclusions

The included studies suggest that staff need more knowledge of dementia and person-centred dementia care and that training intervention studies to enhance staff competence show promising results. However, there is a need for research on the needs of patients with dementia in acute hospital settings regarding physical environment and effect of design elements. There is also a scarcity of intervention studies focusing on the effects of models of care that support the psychosocial needs of patients with dementia.

## Supplementary information

**Additional file 1.**

## Data Availability

The datasets used and/or analysed during the current study are available from the corresponding author on reasonable request.

## Abbreviations

MMAT: The Mixed Methods Appraisal Tool
UK: United Kingdom
